# An Electronic “Tongue” Based on Multimode Multidirectional Acoustic Plate Wave Propagation

**DOI:** 10.3390/s24196301

**Published:** 2024-09-29

**Authors:** Nikita Ageykin, Vladimir Anisimkin, Andrey Smirnov, Alexander Fionov, Peng Li, Zhenghua Qian, Tingfeng Ma, Kamlendra Awasthi, Iren Kuznetsova

**Affiliations:** 1 Kotelnikov Institute of Radio Engineering and Electronics of RAS, Moscow 125009, Russia; ageykin_niki@mail.ru (N.A.); anis@cplire.ru (V.A.); andre-smirnov-v@yandex.ru (A.S.); fionov@cplire.ru (A.F.); 2 State Key Laboratory of Mechanics and Control for Aerospace Structures, College of Aerospace Engineering, Nanjing University of Aeronautics and Astronautics, Nanjing 210016, China; lipeng_mech@nuaa.edu.cn; 3 Shenzhen Research Institute, Nanjing University of Aeronautics and Astronautics, Shenzhen 518063, China; 4 School of Mechanical Engineering and Mechanics, Ningbo University, Ningbo 315211, China; matingfeng@nbu.edu.cn; 5 Department of Physics, Malaviya National Institute of Technology, Jaipur 302017, Rajasthan, India; kawasthi.phy@mnit.ac.in

**Keywords:** acoustic “tongue”, acoustic waves of higher order, piezoelectric plate, attenuation, taste of liquid, liquid properties, food quality

## Abstract

This paper theoretically and experimentally demonstrates the possibility of detecting the five basic tastes (salt, sweet, sour, umami, and bitter) using a variety of higher-order acoustic waves propagating in piezoelectric plates. Aqueous solutions of sodium chloride (*NaCl*), glucose (*C_6_H_12_O_6_*), citric acid (*C_6_H_8_O_7_*), monosodium glutamate (*C_5_H_8_NO_4_Na*), and sagebrush were used as chemicals for the simulation of each taste. These liquids differed from each other in terms of their physical properties such as density, viscosity, electrical conductivity, and permittivity. As a total acoustic response to the simultaneous action of all liquid parameters on all acoustic modes in a given frequency range, a change in the propagation losses (Δ*S*_12_) of the specified wave compared with distilled water was used. Based on experimental measurements, the corresponding orientation histograms of the Δ*S*_12_ were plotted for different types of acoustic waves. It was found that these histograms for different substances are individual and differ in shape, area, and position of their extremes. Theoretically, it has been shown that the influence of different liquids on different acoustic modes is due to both the electrical and mechanical properties of the liquids themselves and the mechanical polarization of the corresponding modes. Despite the fact that the mechanical properties of the used liquids are close to each other, the attenuation of different modes in their presence is not only due to the difference in their electrical parameters. The proposed approach to creating a multi-parametric multimode acoustic electronic tongue and obtaining a set of histograms for typical liquids will allow for the development of devices for the operational analysis of food, medicines, gasoline, aircraft fuel, and other liquid substances without the need for detailed chemical analysis.

## 1. Introduction

Taste is one of the least understood human senses [1]. How human taste sensations correspond to the chemical composition of a substance varies greatly from person to person and in many ways depends on how the brain processes information. However, there are currently five main tastes: sweet, salty, bitter, sour, and umami. Each of these tastes corresponds to a specific chemical substance: glucose, salt, sagebrush, citric acid, and monosodium glutamate. Aqueous solutions of these substances are commonly used as model objects in the development of electronic taste sensors. Work on the creation of such devices has been ongoing since the early 1990s. At first, the question of whether a particular taste corresponded to the electronic response of the sensor was not even considered. Researchers studied the use of gas sensors to determine the quality of food [2]. It was noted that it was difficult to separate the effects of smell and taste for the same substance using purely chemical sensors, and it was necessary to use special neuromorphic algorithms [3,4]. Later, the approaches used to create an “electronic nose” were proposed for the development of an “electronic tongue” [5]. Over the past 40 years, various physical principles have been proposed for the development of taste sensors, including electrochemical methods such as potentiometry and voltammetry [4,5,6,7,8,9,10], the colorimetricmethod [11,12], the conductometric method [13], and acoustic methods [13,14,15,16].

Electrochemical sensors are commonly used for the analysis of multicomponent liquids. They have several advantages, including well-known performance characteristics, ease of setup, low cost, and simplicity of manufacturing. However, they also have some disadvantages, such as temperature dependence and the absorption of solution components during the analysis process.

One of the most intriguing principles behind the development of taste sensors is the acousto-electronic approach. This method involves the use of acoustic delay lines constructed from piezoelectric materials or structures. By employing interdigital transducers positioned on the surface of a piezoelectric material, an acoustic wave can be both excited and detected. The phase and amplitude of this wave are influenced by the characteristics of a sensor film placed between the transducers. The parameters of this sensor film, such as electrical conductivity, permittivity, viscosity, density, and modulus of elasticity, are altered as a result of interactions with the substance being analyzed. These changes in the film’s properties lead to modifications in the acoustic wave’s characteristics within the “piezoelectric plate—sensor film” system [17]. Many acoustic chemical sensors, both gas [18] and liquid [19], are based on this principle. Various metal oxides [20], polymer and graphene-like composite materials [21,22], as well films made from the mycelium of fungi [23], etc., are used as coatings for gas sensors. The main principle behind selecting these coatings is their ability to selectively react with specific chemicals. In the development of liquid sensors, specific films are used that react selectively with a particular chemical or biological substance in a liquid [14,24,25].

Another approach to creating taste sensors involves creating devices without sensory films. In this case, the properties of the acoustic wave depend on the physical characteristics of the liquid located on the surface of the waveguide. In this case, various types of waves with shear horizontal polarization (SH) are most often used. These include surface acoustic waves (SHSAWs) [26,27], Love waves [28], and Gulyaev–Bleustein waves [29]. In addition, recently, waves have been detected in piezoelectric plates with polarization in the plane of the plate [30]. These waves can also be used to create liquid sensors. The advantage of acoustic sensors is that there is no contact between the measured object and the electrodes, which increases the reliability and durability of the devices.

It should be noted that these acoustic sensors can be used in two modes. In the first case, only one specific wave with a fixed wavelength and frequency is used [19,26,27,29]. This wave is chosen for reasons of the greatest sensitivity of its attenuation or phase velocity to the measured parameter, such as viscosity or electrical conductivity of liquid. In the second case, several waves are used that propagate in the same direction with the same crystallographic orientation of the crystal [30].

**Figure 1:**
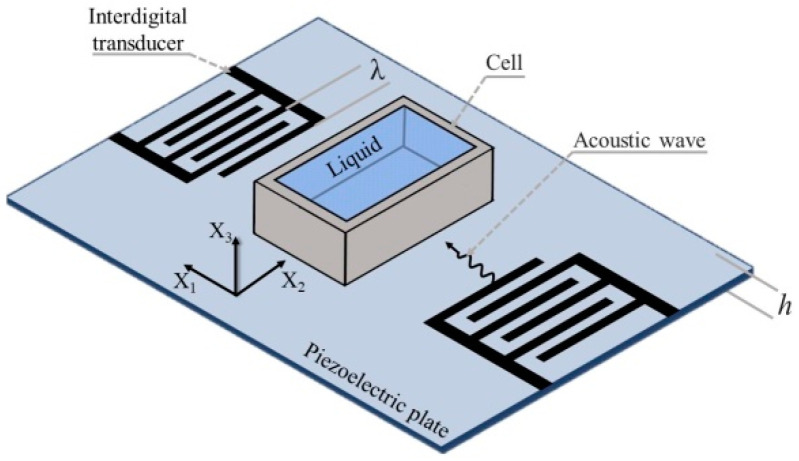
The basic design used in the study of liquid media using acoustic waves in piezoelectric plates.

The basic design of liquid sensors based on these technologies includes a piezoelectric plate, input and output transducers with a period of *λ* for excitation and reception of acoustic waves, and a cell containing the liquid being tested (Figure 1) [17].

The number of acoustic waves that propagate through the plate and interact with the liquid is about 10. During measurements, a phase shift (Δ*φ*), which corresponds to a change in phase velocity (Δ*v*), and a change in wave propagation loss between the input and output transducers (Δ*S*_12_ = Δ*α* × *L*, where Δ*α* and *L* are attenuation change and the length of the liquid cell along the direction of wave propagation) are fixed. The changes Δ*φ* and Δ*S*_12_ are dependent on the viscosity (*η*), density (*ρ*), electrical conductivity (*σ*), permittivity (*ε*), and temperature (*T*) of the liquid (1). Additionally, wave attenuation (*α*) also includes a contribution from *R*(*U_3_*), which is due to the radiation of waves into the liquid due to the presence of a normal displacement component *U_3_*.

(1) $$∆\gamma =\frac{\partial \gamma }{\partial \eta }∆\eta +\frac{\partial \gamma }{\partial \rho }∆\rho +\frac{\partial \gamma }{\partial \sigma }∆\sigma +\frac{\partial \gamma }{\partial \varepsilon }∆\varepsilon +\frac{\partial \gamma }{\partial T}∆T+R(U_{3})$$

$∆\gamma =\frac{\Delta \alpha }{k}+i\times \frac{\Delta \upsilon }{\upsilon_{0}}$ is a complex wavenumber.

The dependences of the values *S*_12_ and *φ* on the viscosity of the liquid are monotonic increasing functions that approach saturation at high values of *η* [30]. The dependencies of *S*_12_ and *φ* on the conductivity of the liquid are bellshaped and exponentially decreasing, respectively. They also reach saturation at high values of *σ* [17]. As the dielectric constant of a liquid increases, the effect of its conductivity on the values of Δ*S*_12_ and Δ*φ* becomes less significant [31]. The dependence of *φ* on temperature *T* can be linear, quadratic, or cubic [32]. Insertion loss (*S*_12_) does not significantly change with temperature (*T*), making it the most suitable parameter for acoustic analysis of liquids [33]. The key properties of the dependencies describedabove are their qualitative similarity in behavior and their quantitative difference in values for different plate waves [33].

Recently, another principle for creating an acoustic liquid sensor has been proposed. This principle is based on using a multimodal approach to implementing a multi-parameter liquid sensor [34]. In this approach, the cell containing the liquid under investigation is placed in the center of a piezoelectric plate, while IDT pairs are positioned around it. By sequentially exciting different types of waves in different crystallographic directions, it was shown that it is possible to construct histograms for liquids with different characteristics. In [35], the possibility of using such a design to create a sensor for basic tastes is demonstrated, and the potential of using machine learning techniques to analyze the obtained data is explored.

The aim of this study was to conduct a theoretical analysis of the impact of liquids used in the experiment on the properties of acoustic waves in a 128°*Y LiNbO_3_* plate. By comparing the experimental findings with theoretical predictions, we aimed to clarify the physical mechanisms of interaction between the studied liquids and different types of high-order acoustic waves, considering their polarization. Based on our theoretical and experimental findings, we developed an algorithm for creating a more effective electronic “tongue” using the propagation of multimode multidirectional acoustic waves in the crystal plate.

## 2. Materials and Methods

### 2.1. Creation of a Multimode Multi-Parameter Liquid Sensor

The multimode multi-parameter liquid sensor was developed on the basis of a 128°*Y LiNbO*_3_ wafer (CQT, Hangzhou, China) with a thickness of 500 μm and a diameter of 2″. On the polished face of the wafer, four delay lines were positioned in a circular arrangement at angles of 0°, 30°, 90°, and 120° to the *X*-axis. These angles were selected based on the maximum anisotropy of this crystallographic orientation, as reported in reference [36]. Each delay line comprised two interdigital transducers (IDTs), with one IDT used for excitation and the other for receiving acoustic waves. In the center of the wafer, a liquid cell was positioned between the IDTs. The liquid cell, made of Teflon, had a diameter, height, and volume of 20 mm, 3 mm, and 764 mm^3^, respectively. Its wall thickness was 1 mm. The cell was bonded to the surface of the plate using solol, and the distance from each IDT to the cell was 1 mm. Each IDT consisted of 40 electrode pairs, with an aperture of 4.7 mm and an operating wavelength of *λ* = 200 μm. The normalized film thickness was *h*/*λ* = 500 μm/200 μm = 2.5. This value provides a large number of acoustic waves along the propagation direction and a variety of mode types. There are 40 pairs of electrodes, which provides a narrow bandwidth for the transducers, approximately 2.5%, and a good frequency resolution for acoustic waves (±0.5 MHz) at close phase velocities *v* (±200 km/s).

The process of creating a set of delay lines on the surface of the plate involved several steps. Initially, the polished surface of the wafer is cleaned using acetone and argon plasma. Then, the sample was coated with an *Al* film (400 nm) using a dc magnetron sputtering system (VSE-PVD-DESKPRO, Vacuum technology and equipment, Novosibirsk, Russia) for approximately 4 min. The discharge power was 200 W, and the working pressure inside the chamber was 5.7 × 10^−3^ Torr. During the sputtering process, the sample was bombarded by high-energy particles and heated, so it was cooled in an Ar atmosphere before leaving the chamber for about 1 h to prevent further heating. The finished aluminum film was coated with a 2 μm thick photoresist SU-8 (Sigma-Aldrich, Darmstadt, Germany) using a desktop centrifuge Sawatec SM-180-BT (SAWATEC AG, Sennwald, Switzerland). The coating was then annealed at 94 °C for 30 min. A digital photo template was created using free software (Layout 6.0), and the mask template was applied to the photoresist-coated sample without a conventional photomask (Smart Print, Microlight 3D, La Tronche, France). The exposure time varied depending on the brightness of the lens, and the exposed areas of the photoresists were then removed using developer P-236 (FRAST-M, Moscow, Russia). The aluminum film in the free areas of the mask was etched with a mixture of orthophosphoric and nitric acids (95:5) for 60 min.

**Figure 2:**
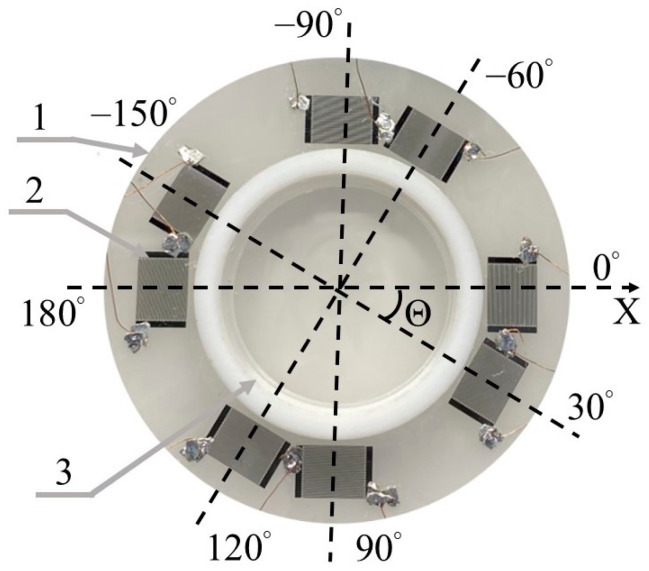
A photo of an experimental setup with four acoustic channels on a single piezoelectric 128°*Y LiNbO_3_* wafer. (1) 128°*Y LiNbO_3_* wafer, (2) interdigital transducers (IDTs), (3) cell for liquid. The angles between the acoustic channels and the crystallographic *X*-axis are *Θ* = 0°, 30°, and 90° и 120°.

The appearance of the experimental sample obtained is shown in Figure 2. Due to the symmetry of the selected cut, the waves propagating at angles of *Θ* = 120°and 60°are identical. For this reason, later in the article, we will consider only *Θ* = 60°.

### 2.2. Measurement Technique

The acoustic propagation losses of the acoustic plate waves between the input and output transducers were selected as the measured acoustic value *S*_12_ = *α* × *L*. The reason for this choice is that this value has a weak dependence on temperature [33]. *S*_12_ measurements were taken in the frequency range of *f* = 10–60 MHz at a room temperature of *T* = 22 ± 1 °C and a normal atmospheric pressure of *p* = 746 mm Hg using a network analyzer, KEYSIGHT 5061B (Keysight, Santa Rosa, CA, USA), with an accuracy of less than ±0.1 dB.

**Figure 3:**
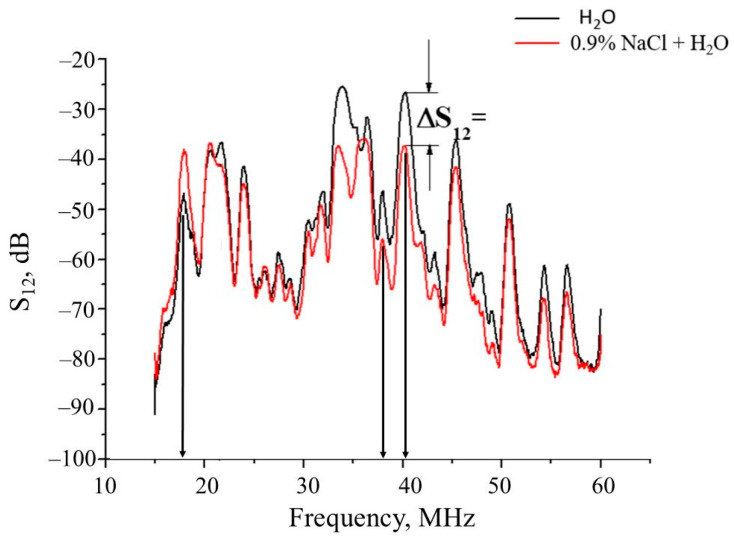
A typical view of the frequency dependence of the insertion losses *S*_12_ of acoustic waves in 128°*Y-X LiNbO_3_* with distilled water (black line) and a 0.9% aqueous *NaCl* (*σ* = 1.4 S/m) solution (red line) in a cell. The arrows indicate some waves with high total losses.

Initially, the frequency dependence of $S_{12}^{H_{2}O}$ was measured in the presence of distilled water (*η* = 1.03 cP, *σ* < 1 mS/m). Then, the test liquid was placed in the cell, and the measurements of the frequency dependence of $S_{12}^{lq}$ were repeated. The acoustic response to the change in the properties of the liquid was represented by the value $∆S_{12}(dB)=S_{12}^{lq}-S_{12}^{H_{2}O}$. In this case, the contribution of radiation losses *R*(*U_3_*) to the response value Δ*S*_12_ is not taken into account. Therefore, different liquids can only be compared based on the total effect of their density, viscosity, electrical conductivity, and dielectric constant on propagation losses (1). As is known, the acoustic field of probing waves penetrates a small liquid depth compared to the thickness of the liquid sample (>1 mm). For water and glycerin, this depth is 0.1 μm and 3 μm, respectively. Therefore, the shape and volume of the liquid sample do not affect the measurement results. In the experiments, 700 ml samples were used. Figure 3 shows the frequency dependence of the insertion loss *S*_12_ measured in one of the acoustic channels of the experimental sample with distilled water (black line) and the tested liquid in a cell (red line).

Figure 3 shows that some acoustic waves respond much more strongly to changes in the properties of the tested liquid compared to other waves. This is due to the different polarizations of these waves and the varying values of their electromechanical coupling coefficients. The calculated values for these parameters for selected waves are given below. Additionally, it should be noted that due to the anisotropy of the material, waves of different types propagating through the liquid at various angles do not experience equal acoustic absorption [17]. From the analysis of the frequency dependences of *S*_12_, the operating frequencies of the acoustic waves that reacted most strongly to a particular liquid were selected. Thus, the measurement of *S*_12_ in the 128°*Y LiNbO_3_* plate for each propagation direction determined by the angle *Θ* was carried out for acoustic waves with frequencies near *f* = 34 ± 0.5 MHz, 37 ± 0.8 MHz, 41 ± 0.5 MHz, 44 ± 1.0 MHz, and 48 ± 0.8 MHz.

The set of responses related to different acoustic waves and channels was presented in the form of histograms. The angle inside each histogram corresponds to the angle *Θ*, and the corresponding value of the Δ*S*_12_ response in dB is depicted along the radius. Different types of acoustic waves were marked with different colors. The histograms for liquids with different tastes were compared based on their shape, area *S*, position, and values of characteristic minima and maxima. The area *S* in dB^2^ was determined by summing the areas of triangles making up each histogram. The sides of these triangles are formed by the responses of neighboring waves ${∆S}_{12}^{\left(n\right)}$ and ${∆S}_{12}^{\left(n+1\right)}$. The angle between these sides is *β* = 360°/*n*, where *n* is the total number of modes used in all channels [35].

(2) $$S=0.5\times \sin\beta \sum \left[{∆S}_{12}^{\left(n\right)}\times {∆S}_{12}^{\left(n+1\right)}\right]$$

Obviously, the greater the acoustic response of a wave, the larger the area of the taste histogram (*S*), and consequently, the higher the cumulative sensitivity of that wave to the overall effect of all the parameters of the liquid being tested.

### 2.3. Liquid Samples Preparation

Aqueous solutions of sodium chloride (*NaCl*), glucose (*C_6_H_12_O_6_*), citric acid (*C_6_H_8_O_7_*), and monosodium glutamate (*C_5_H_8_NO_4_Na*), as well as sagebrush extracts with concentrations of 0.02% and 0.9% were used as test liquids in this study. These substances are known to be responsible for the five basic flavors: salty, sweet, sour, umami, and bitter. To prepare the test solutions, 0.2 g or 9 g of the corresponding substances were dissolved in 1 L of distilled water. The preparation of sagebrush extract was carried out by adding 10 g of dry grass to a glass flask containing 200 mLof distilled water. The solution was then brought to a boil with a closed lid and placed in a boiling water bath for 15 min. After cooling to room temperature, the solution was filtered, and the excess grass was removed by pressing. Finally, 2 μL or 90 μL of the resulting extract was added to 10 ml of distilled water to obtain a sagebrush solution with a concentration of either 0.02% or 0.9%, respectively.

The material constants for the aqueous solutions of sodium chloride (*NaCl*), glucose (*C_6_H_12_O_6_*), citric acid (*C_6_H_8_O_7_*), and monosodium glutamate (*C_5_H_8_NO_4_Na*), which were used in the calculations, are shown in Table 1. The parameters of the sagebrush aqueous solution were measured by our team. Density ($\rho^{lq}$) was measured using a laboratory analytical scale, CAS CAUW-220D (CAS Corp., Seoul, Republic of Korea), and a known volume of liquid. Viscosity ($\eta_{44}^{lq}$) was determined using a selective acoustic sensor [30]. Electrical conductivity ($\sigma_{v}^{lq}$) and dielectric constant ($\varepsilon^{lq}/\varepsilon_{0}$) were measured at 1 MHz using an electrophysical method [37]. The modulus of elasticity ($C_{11}^{lq}$) was measured according to the method described in [17].

### 2.4. Theoretical Method

**Figure 4:**
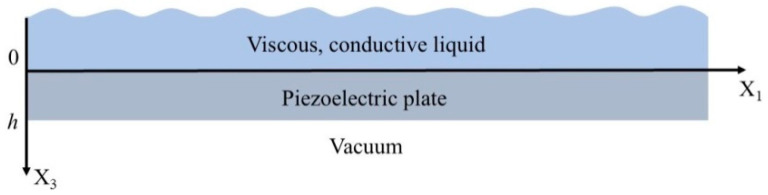
Geometry of the problem.

For theoretical analysis, the structure “piezoelectric plate—viscous and conductive liquid” was considered (Figure 4). The wave propagated in the X_1_ direction.

The piezoelectric plate is bounded by the planes *x*_3_ = 0 and *x*_3_ = *h*. The region with *x*_3_ > *h* corresponds to a vacuum, and the region with *x*_3_ < 0 corresponds to a conductive viscous liquid. The problem is considered two dimensional, so all field components are assumed to be constant in the *x_2_* direction [47]. In order to find the phase velocity, attenuation, and mechanical displacements of the wave, it is necessary to write a system of equations for each medium. For the piezoelectric plate, the equations of motion, Laplace’s equation, and constitutive equations are written as follows [47]:

(3) $$\rho^{pz}\partial^{2}U_{i}^{pz}/\partial t^{2}=\partial T_{ij}^{pz}/\partial x_{j},\partial D_{j}^{pz}/\partial x_{j}=0,$$

(4) $$T_{ij}^{pz}=C_{ijkl}^{pz}\partial U_{l}^{pz}/\partial x_{k}+e_{kij}^{pz}\partial \Phi^{pz}/\partial x_{k},$$

(5) $$D_{j}^{pz}=-\varepsilon_{jk}^{pz}\partial \Phi^{pz}/\partial x_{k}+e_{jlk}^{pz}\partial U_{l}^{pz}/\partial x_{k},$$

For viscous and conductive liquids, the equation of motion, Poisson’s equation, the charge continuity equation, and constitutive equations were used [48].

(6) $$\rho^{lq}\partial^{2}U_{i}^{lq}/\partial t^{2}=\partial T_{ij}^{lq}/\partial x_{j},\partial D_{j}^{lq}/\partial x_{j}=-\delta_{v}^{lq}$$

(7) $$\partial J_{j}^{lq}/\partial x_{j}+\partial \delta_{v}^{lq}/\partial t=0$$

(8) $$T_{ij}^{lq}=C_{ijkl}^{lq}\partial U_{l}^{lq}/\partial x_{k},$$

(9) $$D_{j}^{lq}=-\varepsilon_{jk}^{lq}\partial \Phi^{lq}/\partial x_{k},$$

(10) $$J_{j}^{lq}=-\sigma_{v}^{lq}\partial \Phi^{lq}/\partial x_{j}+d_{v}^{lq}\partial \sigma_{v}^{lq}/\partial x_{j}$$

Here, *U_i_*,*t*, *T_ij_*, *x_j_*, *D_j_*, *Φ*, and *ρ* are the components of the mechanical displacement of particles, time, components of mechanical stress, coordinates, components of electrical displacement, electrical potential, and density, respectively. *C_ijkl_*, *e_ikl_*, and *ε_jk_* are the elastic, piezoelectric, and dielectric constants, respectively. $\delta_{v}^{lq},J_{j}^{lq}$, and $\sigma_{v}^{lq}$ are the volume charge density, components of current density, and bulk conductivity of the liquid, respectively. The indexes *pz* and *lq* indicate that the corresponding variable refers to the piezoelectric film and viscous, conductive liquid, respectively.

In the regions of the vacuum *x*_3_ > *h*, the electrical displacement is satisfied according to Laplace’s equation as follows:

(11) $$\partial D_{j}^{vac}/\partial x_{j}=0,$$

where $D_{j}^{vac}=-\varepsilon_{0}\partial \Phi^{vac}/\partial x_{k}$. Here, *ε*_0_ is the vacuum permittivity, and index *vac* indicates that the variable refers to the vacuum.

Then, the relevant electrical and mechanical conditions at each boundary of the structure, i.e., solid/viscous and conductive liquid and solid/vacuum are written as follows [48]:

(12) $$x_{3}=0:\;U_{i}^{pz}=U_{i}^{lq};T_{i3}^{pz}=T_{i3}^{lq};\Phi^{pz}=\Phi^{lq};D_{3}^{pz}=D_{3}^{lq};\;J_{3}^{lq}=0,$$

(13) $$x_{3}=h:\;T_{i3}^{pz}=0;\Phi^{pz}=\Phi^{vac};D_{3}^{pz}=D_{3}^{vac}.$$

Here, *i* = 1–3, and *h* is the thickness of a piezoelectric film. Due to the viscosity of the liquid considered, the non-zero components of the symmetric complex elastic constants $C_{ij}^{lq*}$ in matrix form will have the following view [48]:

(14) $$\begin{array}{c} C_{11}^{lq*}=C_{22}^{lq*}=C_{33}^{lq*}=C_{11}^{lq}+j\omega \eta_{11}^{lq}\\ C_{12}^{lq*}=C_{13}^{lq*}=C_{23}^{lq*}=C_{11}^{lq}+j\omega \eta_{12}^{lq}\\ C_{44}^{lq*}=C_{55}^{lq*}=C_{66}^{lq*}=j\omega \eta_{44}^{lq} \end{array}$$

where the viscosity of the liquid is accounted as the imaginary part $j\omega \eta_{11}^{lq}$ of the elastic moduli, *j* is an imaginary value, ω = 2πf is the angular frequency, $\eta_{ij}^{lq}$ are the viscosity coefficients in Pa × s, and $\eta_{12}^{lq}=\eta_{11}^{lq}-2\eta_{44}^{lq}$.

The values of phase wave velocity (v) and three partial components of mechanical displacement (*U*_1_, *U*_2_, *U*_3_) in the *x*_3_ = 0 plane were determined using an iterative search procedure based on zeroing the determinant of the matrix representing the corresponding boundary conditions [49]. The material constants for *LiNbO_3_* were taken from a previous study [50] and are presented in Table 2.

In addition, the coefficient of electromechanical coupling for acoustic waves in the frequency range of 33–50 MHz was calculated. This range corresponds to the excitation of acoustic modes with a wavelength of 200 μm at *h*/*λ* = 2.5.

To calculate the electromechanical coupling coefficients (*k*^2^, %), Formula (15) was used [17].

(15) $$k^{2}=200\left(v_{o}-v_{m}\right)/v_{o}$$

where *v*_0_ and *v_m_* are the velocities of acoustic waves for an electrically open plate and an electrically shorted plate on one side, respectively.

## 3. Results and Discussion

### 3.1. Theoretical Results

Table 3, Table 4, Table 5, Table 6 and Table 7 present the results of the theoretical analysis for the structure “128°*Y* + *Θ LiNbO_3_* plate—0.9% aqueous solution of the tested liquid”. It should be noted that, as shown in Figure 3, the presence of a viscous and conductive liquid with different physical properties on the plate surface can cause changes in the absorption and speed of the acoustic wave. The variation in the absorption of the chosen wave as an acoustic response is significant, varying depending on the mode and its polarization and piezoelectric activity. The changes in wave speed are small and result in minor changes to the central frequency of the mode between 0.5 and 0.8 MHz. This allows for measurements to be conducted using a reference liquid (water) and the tested liquid at the same frequency for each mode, eliminating the need to measure the operating frequencies separately. Table 3, Table 4, Table 5, Table 6 and Table 7 provide the values of operating frequencies selected for the experiment and used to construct the flavor diagrams.

The analysis of the data obtained showed that the effect of different liquids on different acoustic waves is influenced by both the electrical and mechanical properties of the liquids and the polarization of the respective waves. Despite the similarity in the mechanical properties of the liquids used, the attenuation of various waves in their presence is not solely due to differences in their electrical characteristics. For example, in Table 3, waves 1 (*f*~41 MHz) and 2 (*f*~44 MHz), with *k*^2^ values of 0.42% and 3.25%, respectively, exhibit similar attenuation values in the presence of a 0.9% aqueous solution of *NaCl*. This is surprising, as one would expect the presence of this liquid to cause significant attenuation for wave 2, which has a higher conductivity. However, this effect seems to be related to the different types of wave polarization. The higher-order waves 1 and 2 appear to have quasi-shear and symmetrical polarizations, respectively. In this case, the attenuation due to the shorting of the tangential electric field components is increased by the radiation losses caused by the presence of the mechanical displacement components *U*_1_ and *U*_3_ for wave 1.

In the case of weakly conductive liquids, such as solutions of glucose, sagebrush, or monosodium glutamate, the difference in their dielectric permittivity is an important factor. However, the electromechanical coupling coefficient of the used waves plays a much smaller role compared to their mechanical polarization. In general, it should be noted that for further development of a multi-parametric acoustic taste sensor, it would be beneficial to use a more anisotropic material for the plate, such as an *X*-cut of lithium niobate. This would allow for a more targeted search for modes that selectively respond to the mechanical and electrical properties of the liquid. Additionally, an analysis of the energy flow angle for each selected acoustic wave could be conducted to optimize the design of the device, considering that this factor could lead to increased efficiency in receiving excited acoustic waves and reduced conversion losses.

### 3.2. Experimental Results

As mentioned above, the analysis of Table 1 revealed that the liquids used in the experiment differ primarily in conductivity and permittivity. However, similar values of density and viscosity for different liquids can also contribute similarly to the overall response of waves of various orders Δ*S*_12_, provided these waves are highly sensitive. Such waves, as expressed in Equation (1), correspond to large values of *dγ/dρ* and *dγ/dη*.

**Figure 5:**
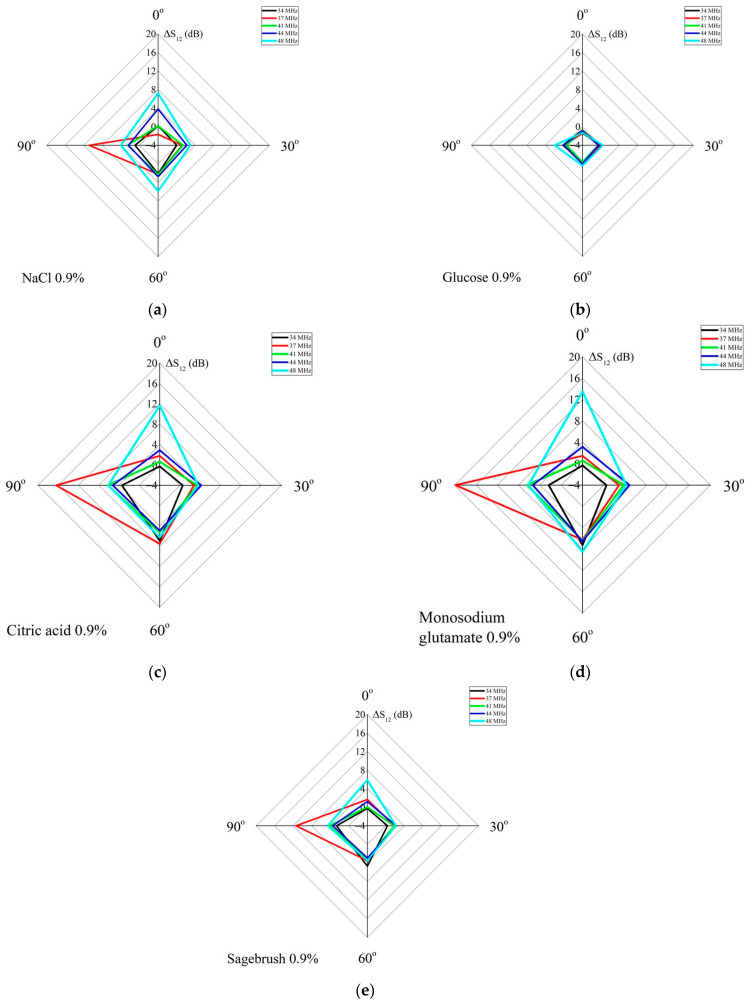
Experimental orientational dependencies of Δ*S*_12_ for acoustic waves with different operating frequencies: ~34 MHz (black line), ~37 MHz (red line), ~41 MHz (green line), ~44 MHz (blue line), and ~48 MHz (light blue line) propagating in a 128°*Y LiNbO_3_* plate with a normalized thickness of *h*/*λ* = 2.5 in the presence of 0.9% aqueous solutions of (**a**) *NaCl*, (**b**) glucose, (**c**) citric acid, (**d**) monosodium glutamate, and (**e**) sagebrush.

Figure 5 shows the histograms of the five base liquids studied, which differ in their physical properties and taste, with a concentration of 0.9%. The responses of the acoustic modes at a given frequency were measured for each liquid, and the results show that they vary depending on the direction of propagation (angle *θ*), as predicted by the theoretical model. It should be noted that due to the difference in the material constants of the piezoelectric plate used in the experiment and data from known sources, it is not possible to conduct an accurate quantitative comparison of theoretical and experimental data. However, the overall qualitative comparison shows a good agreement between theoretical and experimental results.

It can be observed that the constructed experimental taste histograms vary from one another, both in terms of shape and area. The responses from the same samples in different directions can differ several times, as well as the responses from different waves propagating in the same direction. The values of acoustic responses to different tastes reach Δ*S*_12_ = 20 dB, which ensures their reliable measurement with an accuracy of ± 0.1 dB. Analysis has shown that at a concentration of 0.9%, glucose solution exhibits the lowest response for all modes and channels due to its low conductivity and close dielectric constant to that of water.

To compare the obtained histograms, their areas *S* were calculated using Formula (2). The results are presented in Table 8.

Table 8 also presents the average values of the areas of the resulting *S_m_* histograms and their *P_t_* weight ratios for each taste. It is evident that the acoustic responses for different tastes significantly differ from one another. This suggests the potential of the proposed method for developing taste acoustic analyzers.

**Figure 6:**
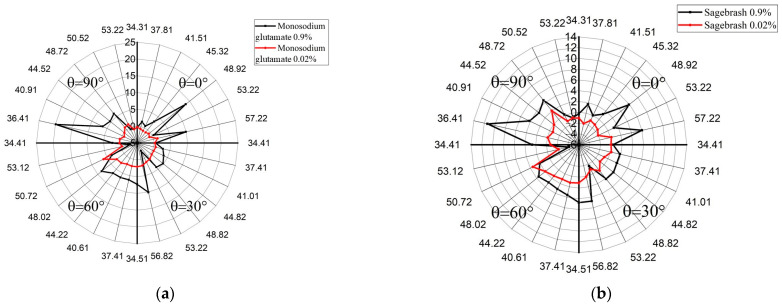
Combined taste histograms of monosodium glutamate (**a**) and sagebrush (**b**) aqueous solutions with concentrations of 0.02% (red line) and 0.9% (black line) measured using the entire set of acoustic modes in a given frequency range and all channels of the 128°*Y LiNbO_3_* plate with a normalized thickness *h*/*λ* = 2.5.

For example, Figure 6 shows the experimental results of measuring Δ*S*_12_ for all acoustic modes in the selected frequency range and all four acoustic channels at *h*/λ = 2.5 in the presence of 0.02% (red line) and 0.9% (black line) aqueous solutions of monosodium glutamate and sagebrush on the surface of the 128°*Y LiNbO_3_* plate.It can be seen that the proposed approach allows you to not only distinguish one taste from another but also to register a change in its concentration.

Table 9, Table 10 and Table 11 present the histograms calculated according to Formula (2) for some food liquids obtained experimentally using the described device. The analysis of the results revealed that these histograms share similar characteristics with those of the five basic flavors. These liquids exhibit high acoustic responses, which depend on the direction of propagation and wave type, and there is an optimal direction with the greatest response difference. This optimal direction is indicated in Table 9, Table 10 and Table 11 in bold.

## 4. Conclusions

This paper shows the possibility of determining the basic tastes (salty, sweet, sour, umami, bitter) using a variety of higher-order acoustic waves in piezoelectric plates. A total of 0.02% and 0.9% aqueous solutions of sodium chloride (*NaCl*), glucose (*C_6_H_12_O_6_*), citric acid (*C_6_H_8_O_7_*), monosodium glutamate (*C_5_H_8_NO_4_Na*), and sagebrush were selected as chemicals for each of the flavors. All these liquids differed from each other in a set of physical properties (density, viscosity, electrical conductivity, permittivity).As a total acoustic response to the simultaneous action of all liquid parameters on all acoustic modes in a given frequency range, a change in the propagation losses (Δ*S*_12_) of the acoustic wave compared with distilled water was used. This parameter is weakly dependent on temperature and strongly depends on the physical properties of the substance being tested. As a result of the experimental studies carried out, it was found that the total response value of Δ*S*_12_ is significant and can reach up to 20 dB compared to distilled water. Based on the experimental measurements, corresponding orientational histograms for Δ*S*_12_ were plotted for various types of acoustic waves. These histograms were found to be unique for different substances, differing in shape, area, and position of extrema.

The theoretical analysis carried out qualitatively confirmed the experimental results. It has been shown that the effect of different liquids on different acoustic waves is due to both the electrical and mechanical properties of the liquids themselves and the polarization of the corresponding waves. Despite the fact that the mechanical properties of the used liquids are close to each other, the attenuation of different waves in their presence is due not only to the difference in their electrophysical parameters.

In general, it should be noted that in order to further develop a multi-parametric acoustic sensor, it would be beneficial to use a more anisotropic material, such as anX-cut of lithium niobate. Itisnecessarytocarryouta more focused theoretical search for specific waves that selectively respond to the mechanical and electrophysical properties of the liquid being analyzed.Additionally, an analysis of the energy flow angle for each selected acoustic wave could be conducted in order to optimize the design of the sensor. By considering this factor, the efficiency of the sensor in receiving excited acoustic waves could be increased, while conversion losses could be reduced.

The proposed approach to creating a multi-parameter, multimode acoustic electronic tongue and generating a set of histograms for various liquids will allow us to develop devices for the efficient analysis of foods, medicines, gasoline, aviation fuel, and other liquids without the need for detailed chemical analysis.

## Figures and Tables

**Table 1:** Physical parameters of 0.9% aqueous solutions of the five base tastes under study.

Physical Parameter	Sodium Chloride (*NaCl*)	Glucose *C_6_H_12_O_6_*	Citric Acid *C_6_H_8_O_7_*	Sagebrush	Monosodium Glutamate *C_5_H_8_NO_4_Na*
$\rho^{lq}$, g/m^3^	1.034 [38]	1.002 [38]	1.0002 [38]	0.999 ± 0.001	0.998 [39]
$\eta_{44}^{lq}$, mP × s	1.085 [38]	1.021 [38]	1.02 [38]	1.01 ± 0.01	1. [40]
$\sigma_{v}^{lq}$, S/m	1.4 [41]	2 × 10^−4^ [41]	0.25 [41]	0	10^−3^ [42]
$\varepsilon^{lq}/\varepsilon_{0}$	77 [43]	79.9 [44]	63.6 [45]	37 ± 2	90 [46]
$C_{11}^{lq}$, 10^9^ Pa	2.25 ± 0.05	2.25 ± 0.05	2.25 ± 0.05	2.25 ± 0.05	2.25 [39]

**Table 2:** Density *ρ* (kg/m^3^), elastic constants *C_ij_* (GPa), piezoelectric coefficients *e_ij_* (C/m^2^), and dielectric permittivity *ε_ij_*/*ε*_0_ of *LiNbO_3_* used in calculations.

		*LiNbO_3_* (*ρ* = 4650)					
$C_{11}^{E}$	$C_{12}^{E}$	$C_{13}^{E}$	$C_{33}^{E}$	$C_{14}^{E}$	$C_{33}^{E}$	$C_{44}^{E}$	$C_{66}^{E}$
203	57.3	75.2	395	8.5104	242.4	59.5	72.8
*e*_15_	*e*_16_	*e*_31_	*e*_33_	*ε*_11_/*ε*_0_	*ε*_33_/*ε*_0_		
3.84	−2.37	0.23	1.3	44.305	27.9		

**Table 3:** Electromechanical coupling coefficient (*k*^2^, %), attenuation normalized to the wavelength (*Γ*, dB/*λ*), and components of mechanical displacement (*U*_1_, *U*_2_, *U*_3_) of acoustic waves in the “128°*Y* + *Θ LiNbO_3_* plate—0.9% aqueous solution of *NaCl*” structures.

*f*, MHz	~34	~37	~41	~44	~48
*Θ* = 0°					
*k*^2^,%	0.03	1.65	0.42	3.25	1.98
*Γ*, dB/*λ*	0.0012	0.0097	0.0045	0.0031	0.044
*U*_1_, *U*_2_, *U*_3_	1; 32; 3	1; 0.03; 0.4	1; 2.5; 0.7	1; 0.05;0.1	1; 0.3; 3.4
*Θ* = 30°					
*k*^2^,%	0.09	1.73	0.82	0.04	1.65
*Γ*, dB/*λ*	0.0006	0.0078	0.0012	0.0019	0.042
*U*_1_, *U*_2_, *U*_3_	1; 2; 0.04	1; 0.5; 0.3	1; 1.7; 0.1	1; 2; 0.4	1; 0.3; 3.2
*Θ* = 60°					
*k*^2^,%	1.48	0.9	0.9	0.3	1.0
*Γ*, dB/*λ*	0.003	0.038	0.0023	0.0032	0.04
*U*_1_, *U*_2_, *U*_3_	1; 0.6; 0.2	1; 4.5; 6.4	1; 5.7; 0.9	1; 1.3; 0.4	1; 0.6; 3.2
*Θ* = 90°					
*k*^2^,%	0.4	1.1	0.4	0.02	1.2
*Γ*, dB/*λ*	0.0023	0.045	0.0023	0.0057	0.045
*U*_1_, *U*_2_, *U*_3_	1; 0; 0.2	1; 0; 1.8	1; 0; 0.2	1; 0; 0.4	1; 0; 6.5

**Table 4:** Electromechanical coupling coefficient (*k*^2^, %), attenuation normalized to the wavelength (*Γ*, dB/*λ*), and components of mechanical displacement (*U*_1_, *U*_2_, *U*_3_) of acoustic waves in the “128°*Y* + *Θ LiNbO_3_* plate—0.9% aqueous solution of glucose” structures.

*f*, MHz	~34	~37	~41	~44	~48
*Θ* = 0°					
*k*^2^,%	0.03	2.24	0.42	3.25	1.98
*Γ*, dB/*λ*	0.001	0.046	0.003	0.0009	0.04
*U*_1_, *U*_2_, *U*_3_	1; 37; 3.8	1; 0.3; 2.9	1; 3.8; 0.9	1; 0.05;0.1	1; 0.05; 0.3
*Θ* = 30°					
*k*^2^,%	0.09	1.95	0.82	0.04	1.65
*Γ*, dB/*λ*	0.005	0.04	0.0006	0.002	0.04
*U*_1_, *U*_2_, *U*_3_	1; 2; 0.04	1; 2.8; 5.2	1; 1.7; 0.1	1; 1.9;0.4	1; 0.3; 3.4
*Θ* = 60°					
*k*^2^,%	0.35	0.9	0.4	0.3	1.
*Γ*, dB/*λ*	0.0016	0.04	0.0013	0.0028	0.038
*U*_1_, *U*_2_, *U*_3_	1; 1.4; 0.2	1; 4.4; 6.3	1; 0.6; 0.2	1; 1.3; 0.4	1; 0.7; 3.2
*Θ* = 90°					
*k*^2^,%	0.4	0.04	0.4	0.02	1.2
*Γ*, dB/*λ*	0.0027	0.0026	0.002	0.0057	0.043
U_1_, *U*_2_, *U*_3_	1; 0; 0.2	1; 0; 0.2	1; 0; 0.2	1; 0; 0.4	1; 0; 6.7

**Table 5:** Electromechanical coupling coefficient (*k*^2^, %), attenuation normalized to the wavelength (*Γ*, dB/*λ*), and components of mechanical displacement (*U*_1_, *U*_2_, *U*_3_) of acoustic waves in the “128°*Y* + *Θ LiNbO_3_* plate—0.9% aqueous solution of citric acid” structures.

*f*, MHz	~34	~37	~41	~44	~48
*Θ* = 0°					
*k*^2^,%	0.03	1.65	0.42	3.25	1.98
*Γ*, dB/*λ*	0.0012	0.017	0.0042	0.013	0.051
*U*_1_, *U*_2_, *U*_3_	1; 34; 3.5	1; 0.03; 0.4	1; 3.4; 0.8	1; 0.05; 0.1	1; 0.3; 3.7
*Θ* = 30°					
*k*^2^,%	0.09	1.73	0.82	3.1	1.65
*Γ*, dB/*λ*	0.0008	0.014	0.0013	0.015	0.046
*U*_1_, *U*_2_, *U*_3_	1; 2; 0.04	1; 0.5; 0.3	1; 1.7; 0.1	1; 0.5;0.3	1; 0.3; 3.4
*Θ* = 60°					
*k*^2^,%	1.48	0.9	0.4	0.3	1
*Γ*, dB/*λ*	0.0073	0.041	0.0025	0.0023	0.042
*U*_1_, *U*_2_, *U*_3_	1; 0.6; 0.2	1; 4.4; 6.3	1; 0.6; 0.2	1; 1.3; 0.4	1; 0.7; 3.3
*Θ* = 90°					
*k*^2^,%	0.4	1.1	0.4	0.02	0.4
*Γ*, dB/*λ*	0.0011	0.049	0.0008	0.0058	0.003
*U*_1_, *U*_2_, *U*_3_	1; 0; 0.2	1; 0; 1.8	1; 0; 0.2	1; 0; 0.4	1; 0; 0.2

**Table 6:** Electromechanical coupling coefficient (*k*^2^, %), attenuation normalized to the wavelength (*Γ*, dB/*λ*), and components of mechanical displacement (*U*_1_, *U*_2_, *U*_3_) of acoustic waves in the “128°*Y* + *Θ LiNbO_3_* plate—0.9% aqueous solution of monosodium glutamate” structures.

*f*, MHz	~34	~37	~41	~44	~48
*Θ* = 0°					
*k*^2^,%	0.03	1.65	0.42	3.25	1.98
*Γ*, dB/*λ*	0.0012	0.0069	0.0032	0.001	0.04
*U*_1_, *U*_2_, *U*_3_	1; 36.6; 3.8	1; 0.03; 0.4	1; 3.7; 0.9	1; 0.05;0.1	1; 0.4; 4
*Θ* = 30°					
*k*^2^,%	0.09	1.73	0.82	3.1	1.65
*Γ*, dB/*λ*	0.0005	0.0053	0.0006	0.0035	0.039
*U*_1_, *U*_2_, *U*_3_	1; 2; 0.05	1; 0.5; 0.4	1; 1.7; 0.1	1; 0.5;0.3	1; 0.3; 3.3
*Θ* = 60°					
*k*^2^,%	1.48	0.9	0.9	0.3	1
*Γ*, dB/*λ*	0.0023	0.036	0.0016	0.0029	0.039
*U*_1_, *U*_2_, *U*_3_	1; 0.6; 0.2	1; 4.8; 6.7	1; 6.5; 1	1; 1.3; 0.4	1; 0.7; 3.2
*Θ* = 90°					
*k*^2^,%	0.4	1.1	0.4	1.2	1.2
*Γ*, dB/*λ*	0.0027	0.042	0.0019	0.042	0.043
*U*_1_, *U*_2_, *U*_3_	1; 0; 0.2	1; 0; 1.8	1; 0; 0.2	1; 0; 3	1; 0; 6.8

**Table 7:** Electromechanical coupling coefficient (*k*^2^, %), attenuation normalized to the wavelength (*Γ*, dB/*λ*), and components of mechanical displacement (*U*_1_, *U*_2_, *U*_3_) of acoustic waves in the “128°*Y* + *Θ LiNbO_3_* plate—0.9% aqueous solution of sagebrush” structures.

*f*, MHz	~34	~37	~41	~44	~48
*Θ* = 0°					
*k*^2^,%	0.03	1.65	0.42	0.14	1.98
*Γ*, dB/*λ*	0.0011	0.0069	0.0028	0.0008	0.04
*U*_1_, *U*_2_, *U*_3_	1; 41; 4.1	1; 0.03; 0.4	1; 3.7; 0.9	1; 24; 2.1	1; 0.4; 4
*Θ* = 30°					
*k*^2^,%	0.09	1.73	0.82	3.1	0.04
*Γ*, dB/*λ*	0.0005	0.0053	0.00068	0.0036	0.0018
*U*_1_, *U*_2_, *U*_3_	1; 2; 0.05	1; 0.5; 0.4	1; 1.7; 0.1	1; 0.5;0.3	1; 1.9; 0.4
*Θ* = 60°					
*k*^2^,%	0.8	0.9	0.4	0.3	1
*Γ*, dB/*λ*	0.047	0.036	0.0013	0.0028	0.038
*U*_1_, *U*_2_, *U*_3_	1; 1; 2.7	1; 4.8; 6.7	1; 0.7; 0.2	1; 1.4; 0.4	1; 0.7; 3.2
*Θ* = 90°					
*k*^2^,%	0.4	1.1	0.4	0.02	0.4
*Γ*, dB/*λ*	0.0027	0.042	0.0019	0.0057	0.0022
*U*_1_, *U*_2_, *U*_3_	1; 0; 0.2	1; 0; 1.8	1; 0; 0.2	1; 0; 0.36	1; 0; 0.2

**Table 8:** Histogram areas of 0.9% aqueous solutions of basic tastes.

*f*, MHz	~34	~37	~41	~44	~48	S_m_	P_t_
NaCl (salty taste)	155.43	232.42	184.15	231.91	334.21	227.62	1.89
Glucose (sweet taste)	119.59	115.78	110.41	117.08	138.92	120.356	1
Citric acid (acidic taste)	225.97	444.42	281.01	303.13	435.53	338.012	2.8
Monosodium glutamate (umami)	216.30	459.34	300.23	335.77	500.78	362.484	3.011
Sagebrush (bitter taste)	194.36	309.60	210.35	218.76	287.53	244.12	2.02

**Table 9:** Histogramareas of twovarieties of drinkingwater.

*Θ*	*S*_1_ *H_2_O* Sant Spring	*S*_2_ *H_2_O* Aquanika
0°	198 (1)	178 (0.9)
30°	279( 1)	258 (0.92)
60°	236 (1)	214 (0.9)
**90°**	219 **(1)**	307 **(1.4)**

**Table 10:** Histogram areas of three types of spirits.

*Θ*	*S*_1_ Vodka Tscarskaya Gold (Russia)	*S*_2_ Dark Rum Rhea (India)	*S*_3_ Cognac Old Kenigsberg (Russia)
0°	155 (1)	157 (1.01)	195 (1.26)
**30°**	252 **(1)**	655 **(2.6)**	1000 **(4)**
60°	179 (1)	219 (1.22)	244 (1.36)
90°	208 (1)	237 (1.14)	246 (1.18)

**Table 11:** Histogram areas of two types of wine.

*Θ*	*S*_1_ White Wine Risling High Coast (Kuban, Russia)	*S*_2_ Red Wine Merlo High Coast (Kuban, Russia)
0°	365 (1)	448 (1.22)
**30**°	519 **(1)**	673 **(1.3)**
60°	548 (1)	590 (1.07)
90°	916 (1)	1035 (1.13)

## Data Availability

Data are contained within the article.

## References

[1] Holmes B. (2017). Flavor: The Science of Our Most Neglected Sense.

[2] Hoffheins B.S., Lauf R.J. (1990). Use of chemical sensor arrays for food and fragrance analysis. J. Sens. Stud..

[3] Nakamoto T., Fukuda A., Moriizumi T. (1993). Perfume and flavor identification by odour sensing system using quartz-resonator sensor array and neural-network pattern recognition. Sens. Actuators B Chem..

[4] Di Natale C., Paolesse R., Macagnano A., Mantini A., D’Amico A., Ubigli M., Legin A., Lvova L., Rudnitskaya A., Vlasov Y. (2000). Application of a combined artificial olfaction and taste system to the quantification of relevant compounds in red wine. Sens. Actuators B Chem..

[5] Winquist F. (2008). Voltammetric electronic tongues - Basic principles and applications. Microchim. Acta.

[6] Nakata S., Takemura K., Neya K. (2001). Chemical sensor based on nonlinearity: Principle and application. Anal. Sci..

[7] Ha D., Sun Q., Su K., Wan H., Li H., Xu N., Sun F., Zhuang L., Hu N., Wang P. (2015). Recent achievements in electronic tongue and bioelectronic tongue as taste sensors. Sens. Actuators B Chem..

[8] Ciosek P., Chudy M., Górski Ł., Grabowska I., Grygoowicz-Pawlak E., Malinowska E., Wróblewski W. (2009). Potentiometric studies and various applications of solid state electrodes based on silicon and epoxy glass structures—An overview. Electroanalysis.

[9] Kachoosangi R.T., Wildgoose G.G., Compton R.G. (2008). Carbon nanotube-based electrochemical sensors for quantifying the ‘heat’ of chilli peppers: The adsorptive stripping voltammetric determination of capsaicin. Analyst.

[10] Cetó X., Pérez S., Prieto-Simón B. (2022). Fundamentals and application of voltammetric electronic tongues in quantitative analysis. TrAC Trends Anal. Chem..

[11] Chung S., Park T.S., Park S.H., Kim J.Y., Park S., Son D., Bae Y.M., Cho S.I. (2015). Colorimetric sensor array for white wine tasting. Sensors.

[12] Shen J., Li T., Chen Y., Zhou H., Dong S., Wei Y., Li F., Ning J., Li L. (2024). Tracing the geographic origin of CTC black tea based on colorimetric sensor array response to taste substances combined with chemometrics. Food Control.

[13] Śliwińska M., Wiśniewska P., Dymerski T., Namieśnik J., Wardencki W. (2014). Food analysis using artificial senses. J. Agric. Food Chem..

[14] Dizon M., Tatarko M., Hianik T. (2020). Advances in analysis of milk proteases activity at surfaces and in a volume by acoustic methods. Sensors.

[15] Sehra G., Cole M., Gardner J.W. (2004). Miniature taste sensing system based on dual SH SAW sensor device: An electronic tongue. Sens. Actuators B Chem..

[16] Cole M., Spulber I., Gardner J.W. (2015). Surface acoustic wave electronic tongue for robust analysis of sensory components. Sens. Actuators B Chem..

[17] Balantine D.S., White R.M., Martin S.J., Ricco A.J., Zellers E.T., Frye G.C., Wohltjen H. (1996). Acoustic Wave Sensors: Theory, Design and Physico-Chemical Applications.

[18] Panneerselvam G., Thirumal V., Pandya H.M. (2018). Review of surface acoustic wave sensors for the detection and identification of toxic environmental gases/vapours. Arch. Acoust..

[19] Lange Lange K., Rapp B.E., Rapp M. (2008). Surface acoustic wave biosensors: A review. Anal. Bioanal. Chem..

[20] Li X., Sun W., Fu W., Lv H., Zu Z., Guo Y., Gibson D., Fu Y.-Q. (2023). Advances in sensing mechanisms and micro/nanostructured sensing layers for surface acoustic wave-based gas sensors. J. Mater. Chem. A.

[21] Wang B., Zhou L., Wang X. (2023). Surface acoustic wave sensor for formaldehyde gas detection using the multi-source spray-deposited graphene/PMMA composite film. Front. Mater..

[22] Lim Y.M., Leong A., Yap K.Z., Swamy V., Ramakrishnan N. (2024). Deep learning approach to estimate relative humidity contribution in VOC response of LCM-graphene oxide-based VOC sensors. IEEE Sens. J..

[23] Smirnov A., Anisimkin V., Krasnopolskaya L., Guliy O.I., Sinev I., Simakov V., Golyshkin A., Almyasheva N., Ageykin N.A., Kuznetsova I.E. (2023). Features of the formation of sensitive films based on mycelium of higher fungi for surface and plate acoustic waves gas sensors. Sensors.

[24] Zhang Q., Tan L.F., Chen Y.X., Zhang T., Wang W.J., Liu Z., Fu L. (2016). Human-like sensing and reflexes of graphene-based films. Adv. Sci..

[25] Pang J., Peng S., Hou C., Wang X., Wang T., Cao Y., Zhou W., Sun D., Wang K., Rummeli M.H. (2023). Applications of MXenes in human-like sensors and actuators. Nano Res..

[26] Kondoh J., Nakayama K., Kuznetsova I. (2021). Study of frequency dependence of shear horizontal surface acoustic wave sensor for engine oil measurements. Sens. Actuators A Phys..

[27] Adetula O., Aimofumhe E., Badewole F., Ijale C., Thomas M. (2023). Sensitivity measurements for a 250 MHz quartz shear-horizontal surface acoustic wave biosensor under liquid viscous loading. AIP Adv..

[28] Choudhari A., Rube M., Sadli I., Sebeloue M., Tamarin O., Dejous C. (2024). Love-wave acoustic sensors behavior in complex liquids: Multiparameter sensing using acoustic and electrical signals. IEEE Sens. J..

[29] Kiełczyński P., Szalewski M., Balcerzak A., Rostocki A.J., Tefelski D.B. (2011). Application of SH surface acoustic waves for measuring the viscosity of liquids in function of pressure and temperature. Ultrasonics.

[30] Anisimkin V., Shamsutdinova E., Li P., Wang B., Zhu F., Qian Z., Kuznetsova I. (2022). Selective detection of liquid viscosity using acoustic plate waves with in-plane polarization. Sensors.

[31] Martin S.J., Ricco A.J., Nimczyk T.M., Frye G.C. (1989). Characterization of SH acoustic plate mode liquid sensor. Sens. Actuators.

[32] Anisimkin V.I., Voronova N.V., Pucjkov Y.V. (2015). General properties of the acoustic plate modes at different temperatures. Ultrasonics.

[33] Anisimkin V., Kolesov V., Kuznetsova A., Shamsutdinova E., Kuznetsova I. (2021). An analysis of the water-to-ice phase transformation using acoustic plate waves. Sensors.

[34] Anisimkin V.I., Voronova N.V. (2017). Integral array of acoustic sensors for micro-liter liquid discrimination. Bull. Russ. Acad. Sci. Phys..

[35] Ageykin N.A., Anisimkin V.I., Voronova N.V., Telminov O.A., Shamin E.S. (2024). Measurement and processing of the acoustic Lamb wave responses towards water solutions of basic flavors. Radioelektron. Nanosist. Inf. Tehnol..

[36] Slobodnik A.J., Conway J.R., Delmonico E.D. (1973). Microwave Acoustic Handbook, V.1A, Surface Wave Velocities.

[37] Shamsutdinova E.S., Anisimkin V.I., Fionov A.S., Smirnov A.V., Kolesov V.V., Kuznetsova I.E. (2023). Improvement of methods for studying the electrophysical viscous properties of liquids. Acoust. Phys..

[38] Lide D.R. (2000). Handbook of Chemistry and Physics.

[39] Firdaus A.A., Chakraborty N., Juglan K.C. (2024). Thermo-acoustical investigation of monosodium glutamate food preservative in an aqueous solution of poly-ethylene glycols (400 and 600) by using ultrasonic technique. Chem. Thermodyn. Therm. Anal..

[40] Mougin P., Wilkinson D., Roberts K.J., Jack R., Kippax P. (2003). Sensitivity of particle sizing by ultrasonic attenuation spectroscopy to material properties. Powder Technol..

[41] Weast R.C., Astle M.J. (1985). Chemical Rubber Company Handbook of Chemistry and Physics.

[42] Apelblat A., Manzurola E., Orekhova Z. (2007). Electrical conductance studies in aqueous solutions with glutamic ions. J. Solut. Chem..

[43] Peyman A., Gabriel C., Grant E.H. (2007). Complex permittivity of sodium chloride solutions at microwave frequencies. Bioelectromagnetics.

[44] Yoon G. (2011). Dielectric properties of glucose in bulk aqueous solutions: Influence of electrode polarization and modeling. Biosens. Bioelectron..

[45] Routray W., Orsat V. (2014). Variation of dielectric properties of aqueous solutions of ethanol and acids at various temperatures with low acid concentration levels. Phys. Chem. Liq. Int. J..

[46] Bordi F., Cametti C., Paradossi G. (1996). A comparative study of the high-frequency dielectric properties of poly (α-Glutamate) and poly (γ-Glutamate) aqueous solutions. Pept. Sci..

[47] Adler E.L., Slaboszewicz J.K., Farnell G.W., Jen C.K. (1990). PC software for SAW propagation in anisotropic multilayers. IEEE Trans. Ultrason. Ferroelectr. Freq. Control.

[48] Zaitsev B.D., Kuznetsova I.E., Joshi S.G., Borodina I.A. (2001). Acoustic waves in piezoelectric plates bordered with viscous and conductive liquid. Ultrasonics.

[49] Kuznetsova I.E., Zaitsev B.D., Joshi S.G., Teplykh A. (2007). Effect of a liquid on the characteristics of antisymmetric lamb waves in thin piezoelectric plates. Acoust. Phys..

[50] https://www.bostonpiezooptics.com/lithium-niobate.

